# ALOHA: developing an interactive graph-based visualization for dietary supplement knowledge graph through user-centered design

**DOI:** 10.1186/s12911-019-0857-1

**Published:** 2019-08-08

**Authors:** Xing He, Rui Zhang, Rubina Rizvi, Jake Vasilakes, Xi Yang, Yi Guo, Zhe He, Mattia Prosperi, Jinhai Huo, Jordan Alpert, Jiang Bian

**Affiliations:** 1 Department of Health Outcomes and Biomedical Informatics, College of Medicine, University of Florida, Gainesville, FL USA; 2 Institute for Health Informatics and College of Pharmacy, University of Minnesota, Minneapolis, MN USA; 3 Cancer Informatics and eHealth Core, University of Florida Health Cancer Center, Gainesville, FL USA; 4 School of Information, Florida State University, Tallahassee, FL USA; 5 Epidemiology, University of Florida, Gainesville, FL USA; 6 Department of Health Services Research, Management and Policy, University of Florida, Gainesville, FL USA; 7 Department of Advertising, College of Journalism and Communications, University of Florida, Gainesville, FL USA

**Keywords:** Knowledge base, Knowledge graph, User-centered design, Usability, Dietary supplement, Online health information

## Abstract

**Background:**

Dietary supplements (DSs) are widely used. However, consumers know little about the safety and efficacy of DSs. There is a growing interest in accessing health information online; however, health information, especially online information on DSs, is scattered with varying levels of quality. In our previous work, we prototyped a web application, ALOHA, with interactive graph-based visualization to facilitate consumers’ browsing of the integrated DIetary Supplement Knowledge base (iDISK) curated from scientific resources, following an iterative user-centered design (UCD) process.

**Methods:**

Following UCD principles, we carried out two design iterations to enrich the functionalities of ALOHA and enhance its usability. For each iteration, we conducted a usability assessment and design session with a focus group of 8–10 participants and evaluated the usability with a modified System Usability Scale (SUS). Through thematic analysis, we summarized the identified usability issues and conducted a heuristic evaluation to map them to the Gerhardt-Powals’ cognitive engineering principles. We derived suggested improvements from each of the usability assessment session and enhanced ALOHA accordingly in the next design iteration.

**Results:**

The SUS score in the second design iteration decreased to 52.2 ± 11.0 from 63.75 ± 7.2 in our original work, possibly due to the high number of new functionalities we introduced. By refining existing functionalities to make the user interface simpler, the SUS score increased to 64.4 ± 7.2 in the third design iteration. All participants agreed that such an application is urgently needed to address the gaps in how DS information is currently organized and consumed online. Moreover, most participants thought that the graph-based visualization in ALOHA is a creative and visually appealing format to obtain health information.

**Conclusions:**

In this study, we improved a novel interactive visualization platform, ALOHA, for the general public to obtain DS-related information through two UCD design iterations. The lessons learned from the two design iterations could serve as a guide to further enhance ALOHA and the development of other knowledge graph-based applications. Our study also showed that graph-based interactive visualization is a novel and acceptable approach to end-users who are interested in seeking online health information of various domains.

## Background

A large body of evidence shows that some dietary supplements (DSs) are beneficial for overall health, and in some cases, can help manage certain health conditions [1, 2]. With the increase in health awareness, the past few decades have witnessed a rapid growth of DS use in the United States. The proportion of US adults using at least one DS has increased from 42% in 1988–1994 to 54% in 2003–2006 [3, 4]; while in 2011–2012, roughly 52% of US adults reported having used a DS in the past 30 days [4]. Consumers are thus increasingly interested in learning about DS products and DS-related health information [5, 6]. However, most consumers have limited, even erroneous knowledge about DSs, especially their safety and efficacy. Consumers often believe that DSs are held to the same safety and efficacy standards as over-the-counter medications. On the contrary, DSs are not regulated as drugs and are not required to undergo rigorous designed clinical trials. Further, approval from the US Food and Drug Administration (FDA) before the sale in the US is not necessary for a DS, unless the product is intended for therapeutic use [7].

Meanwhile, because of the rapid growth of the internet, recent years has witnessed an increasing trend of online health information seeking [8]. For many people, the internet is the first place to find health information [9, 10]. The public is also increasingly interested in online DS-related health information. Nevertheless, many studies have shown that both health professionals and the general public have difficulty in finding trusted, high-quality, and easy-to-understand online health information in general [11, 12]. Our study [13] found that online weight loss information returned by search engines such as Google often rank less reliable sites higher than those of better/quality sites, and often top-ranked sites include advertisements that make unrealistic weight loss promises. And more specifically, both the quality and accessibility of DS-related online health information are worrisome [14, 15]. A recent study shows that with the emergence of new online media platforms and the popularity of social media, consumers face new challenges in consuming online health information [16]. The study revealed several problems in web pages that contain both conventional evidence-based treatments (e.g., healthy balanced diet exercise) and unconventional treatments (e.g., DSs): 1) most pages either promised or strongly suggested that there was a high likelihood of complete recovery from diseases; 2) the background and credentials of the authors and the information sources they cited (if any) vary widely; 3) many pages sold commercial products including DSs and books; 4) these pages often use a large number of personal emotional anecdotes and actively referred to the word cure; 5) most pages present some biological explanations of the treatments, and some of the explanations involve levels of complexity far beyond the level of educated public consumers [16]. Considering that consumers frequently turn to online health information resources, it is crucial to assist them in obtaining evidence-based, high-quality online health information rather than information that is controversial, exaggerated, or not evidence-based.

In the past few decades, a large number of research studies ranging from in vitro to in vivo experiments, from animal models to human trials and from anectodical case reports to randomized controlled trials have generated a tremendous amount of data and information related to DSs. However, this rich information needs to be well organized and translated into usable knowledge before consuming. Also, their quality should have been assessed and validated by experts [17]. There are many well-known, high-quality, evidence-based electronic resources for DS information, including commercial databases such as Natural Medicines (NM) [18], as well as public databases such as U.S. Dietary Supplement Label Database (DSLD) [19], Canadian Natural Health Product Ingredient Database (NHPID) [20], Licensed Natural Health Products Database (LNHPD) [21], and Memorial Sloan Kettering Cancer Center’s (MSKCC) About Herbs database [22]. Nevertheless, there is still a critical need to link heterogenous DS information across these different resources via a controlled vocabulary and a standard data model [23]. Further, very limited safety information exists in these resources. To address these problems, iDISK was created with a standardized DS ontology model [24] integrating information from four high-quality scientific resources (i.e., DSLD, NHPID, LNHPD, and MSKCC’s About Herbs, that focuses on capturing and representing essential information about DS ingredients, primarily their interactions with drugs, and potential severe adverse events. iDISK can be easily integrated with other resources in the Unified Medical Language System (UMLS) as it follows its Rich Release Format (RRF). Nevertheless, there is not yet a solution for general consumers to access high-quality scientific knowledge in the iDISK knowledge base (KB) with a user-friendly interface.

Visualization is necessary to organize and facilitate navigation of massive information to inspire “visual thinking” [25]. A well-designed interactive visualization system can facilitate users’ understanding and consumption of the information [26]. Our previous work on visualizing social networks has shown the potential to help people explore, perceive, and reason with graph-structured data [27]. Ontologically-structured knowledge bases (or knowledge graphs) such as iDISK can be naturally visualized as graphs/networks (i.e., nodes connected with links). We believe that a graph-based interactive visualizations of the semantic search results from iDISK can help users query the knowledge base efficiently in order to find and understand DSs and their related essential information.

In our previous work [28], we prototyped a web-based application with interactive graph-based visualization, named ALOHA (i.e., dietAry suppLement knOwledge grapH visuAlization), to facilitate browsing of the iDISK KB. In this work, we further refined ALOHA following a user-centered design (UCD) approach to improve end-users’ user experience (UX). Built upon our previous study [28], we carried out two new UCD iterations aiming to increase the usability and UX of ALOHA. For each of the two design iterations, we first analyzed the feedback and System Usability Scale (SUS) scores collected from previous design session, refined existing functionalities and added necessary new features, and further evaluated the usability of ALOHA with a group of intended end-users.

The rest of the paper is organized as follows. We will first introduce iDISK KB, the application architecture of ALOHA and our UCD process in the “Methods” section. The usability testing results for each design iteration and the key features updated in ALOHA will be presented in the “Results” section. We will discuss the lessons learned, summarize current work, and propose future directions in the “Discussion and Conclusions” section.

## Methods

### Data sources

Dietary supplements, often defined as a category of food, are widely consumed in people’s daily life, despite the limited knowledge around their safety and efficacy as well as the lacking of any well-established regulatory policies, unlike their drug counterparts [23]. There is an urgent need for a well-integrated, evidence-based DS knowledge base that can facilitate dissemination of scientific knowledge around DS use. The iDISK was created as a standardized source of DS and DS related safety information (with a focus on drug-supplement interactions [DSIs]) to help clinicians, researchers, and consumers make informed decisions about DS use. The data in iDISK are standardized in an ontological structure consisting of three main parts: concepts related to DSs, relationships between these concepts, and the attributes of these concepts and relationships. The current version of iDISK focuses on information around individual DS ingredients and their relationships with 4 other concept categories: 1) drugs or herbs with which the DS ingredients will interact (e.g., “*Melatonin interacts with Nifedipine*”), 2) diseases or conditions on which the DS ingredients will affect (e.g., “*Melatonin is effective for insomnia or sleeplessness*”), 3) signs/symptoms through which DS ingredients manifest their adverse reactions (e.g., “*Melatonin has adverse effects of tachycardia or increase heart rate*”); and 4) DS products that contain the DS ingredients (e.g., “*Sleepaid contains the Melatonin”).*

### Development of ALOHA following a user-centered design process

**Fig. 1:**
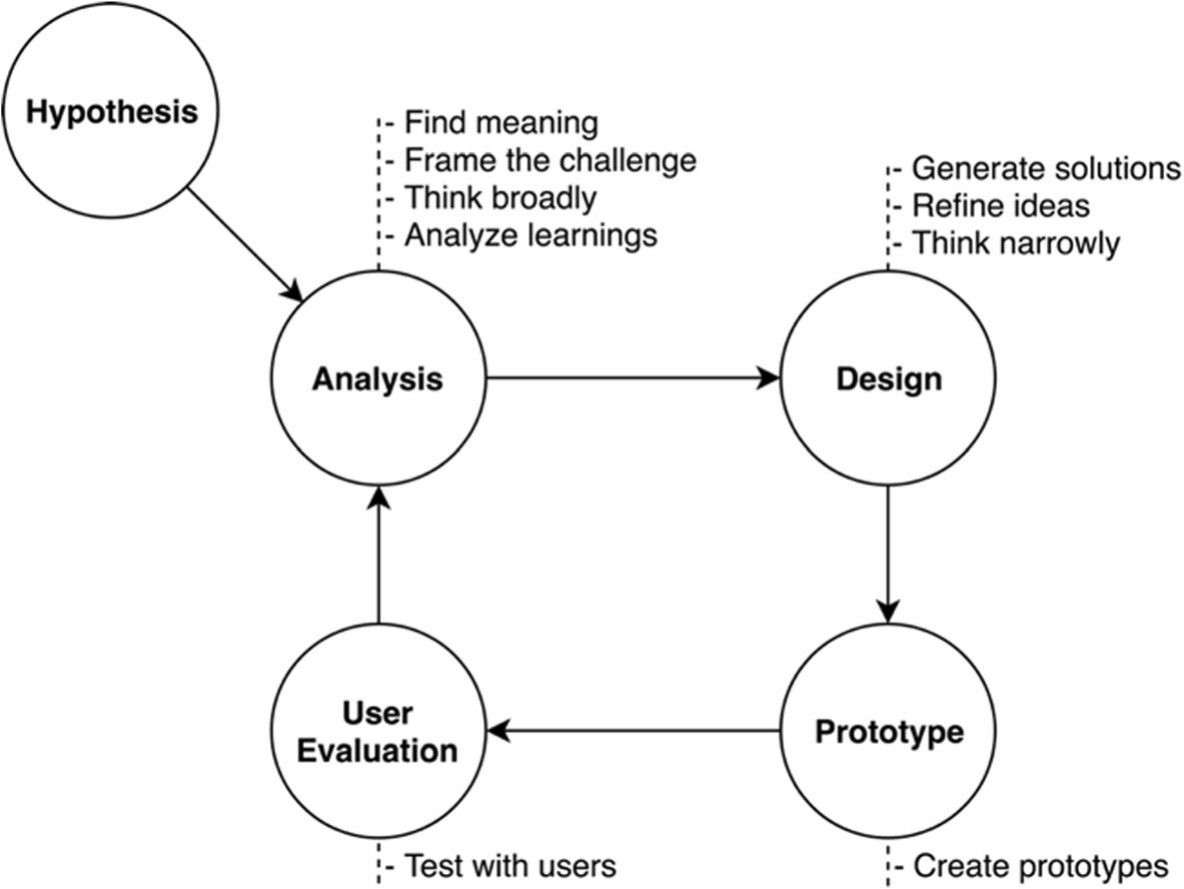
An iterative user-centered design process for developing ALOHA

The main goal of ALOHA is to facilitate the target users of iDISK to find and consume DS information, especially safety information associated with DS ingredients. UX is one of the most critical factors to be considered. Thus, we employed a UCD process in developing ALOHA. As shown in Fig. 1, our design and development process of ALOHA can be divided into 5 steps: 1) an initial hypothesis making process through summarizing existing research; 2) analysis of the needs and requirements of the intended end-users; 3) a prototype design with the required functionalities and related visualizations; 4) a working prototype development; and 5) usability assessments and collection of user feedback. The last four steps: analysis, design, prototype, and user evaluation should be conducted iteratively.

#### Hypothesis making

A project following UCD principles should begin with a hypothesis that has come from stakeholders and was informed by existing literature. Based on our prior work on visualizing social networks [27], discussions with various domain experts including clinicians, pharmacists, DS researchers, ontologists and experts on semantic web knowledge base/graph, as well as our review of existing literature, we hypothesized that a graph-based visualization would be an effective way for end-users to explore, perceive, and reason over the graph-structured knowledge presented in iDISK.

#### Analysis of the needs and requirements

In our previous work [28], which was the first design iteration, we gathered a list of potential DS relevant questions that would interest the intended end users from relevant Yahoo Answers! (i.e., a social question and answer system) posts (e.g., searching for DS products to remedy specific conditions and questions on potential safety issues of the DS products). Based on these consumer questions, we identified an initial collection of intended end users’ needs and requirements. In the second and third design iteration, we analyzed the feedback collected from previous iteration’s usability assessment session and updated the needs and requirements (e.g., adding additional functionalities to help end-users explore product-level information such as “*What ingredients does product X contain*?”).

#### Design

In the first design iteration, we reviewed existing literature on visualization approaches in presenting ontologically-structured data and determined that graph−/ network-based visualizations have been widely used and proven useful. We have also identified a number of existing graph-based visualization frameworks such as D3.js [29] and Sigma.js [30] as well as those that are embedded in state-of-the-art graph databases (e.g., Neo4j [31] and GraphDB [32]). We then sketched the initial features desired by the intended end-users and developed the functional requirements based on the needs and requirements. In the following two design iterations, based on user feedback from the usability assessment sessions, we redesigned or refined existing features (e.g., removed unnecessary information that clogged user attentions such as the UMLS semantic type of DS ingredients) as well as added a number of new features (e.g., added “Zoom and Filters” to help user focus on the information that is more important to the specific user). One key design principle that we consistently followed throughout our UCD iterations is that the user interface (UI) should be clean and simple yet provide all the necessary functionalities. When conflicts arose between the two (clean vs. comprehensive), we chose a simpler design that makes the UI clean.

#### Application

**Fig. 2:**
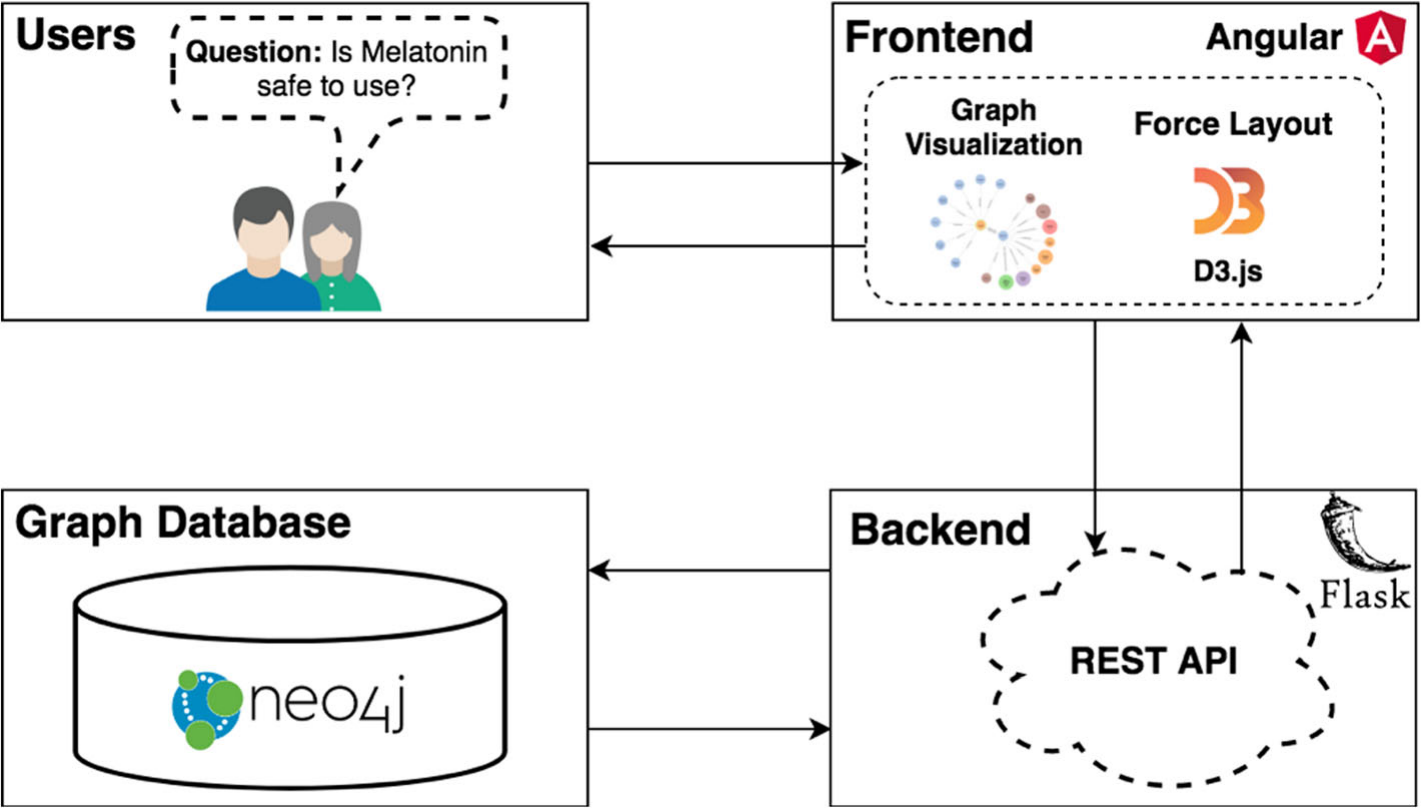
The system architecture of ALOHA

As depicted in Fig. 2, ALOHA includes three components:

1. A Neo4j graph database that can store ontologically-structured knowledge and execute semantic queries. We used Neo4j as the graph database for ALOHA. Data in iDISK are in UMLS RRF format that cannot be imported into Neo4j directly. Thus, we first transformed the RRF formatted data into comma-separated values (CSV) format and then imported into a Neo4j database via Cypher [33]—a graph query language designed specifically for Neo4j.
2. A Flask-based Python backend with Representational State Transfer-ful (RESTful) application programming interfaces (APIs). We used the Flask framework [34], a popular web application framework in Python, as the backend framework. We followed the best practices in developing web applications and provided RESTful—an architectural style for designing web services—API endpoints to connect the frontend UI with the backend graph-based data services through the Neo4j Python driver [35].
3. A web frontend built with the popular Angular web application framework with d3.js-powered interactive visualizations. We used Angular [36] (commonly referred to as “Angular 2+” or “Angular v2 and above”)—a TypeScript-based [37] open-source front-end web application framework for rapid application development combined with d3.js-based interactive graph visualizations. We chose a force-directed graph drawing algorithm to provide an aesthetically-pleasing visualization of the information in iDISK. A force-directed layout uses a physics-based simulator for positioning visual elements. In a force-directed graph layout, there are two types of forces: a repulsive charge force and a pseudo-gravity force. Forces can be set up between nodes, so that 1) all nodes repel one another; 2) nodes are attracted to the center of the gravity; 3) linked nodes are a fixed distance apart; and 4) nodes may not overlap. A force-directed layout keeps nodes centered in the visible area and avoids expulsion of disconnected subgraphs.

As shown in Fig. 2, as users interact with the frontend interface (e.g., post their DS questions materialized as interactions with the graph-based visualizations), the frontend sends HTTP requests to the various REST API end-points on the backend. The backend services will reformulate the questions and user interactions as Neo4j Cypher queries through various pre-defined query templates and then retrieve graph-structured data from the Neo4j graph database with reasoning enabled. The Cypher query results are processed on the Flask backend (mostly house-keeping procedures such as reformatting the data into JavaScript Object Notation, JSON, so that the data can be easily consumed by d3.js). The processed data will be returned to the frontend, where the d3.js-powered interactive visualization module will render the nodes and links according to data received, as our query results are essentially a subgraph of the iDISK knowledge base relevant to the specific user questions.

#### User acceptance and usability assessments

In each design iteration, we inspected the usability of ALOHA with a focus group of 8 to 10 participants recruited from a convenience sample (i.e., college students). Our primary research questions were: (1) Will users accept the graph-based visualization of DS information? What are facilitators and barriers to the acceptance? (2) Are existing question templates useful? What other ways do the users want to facilitate navigation of the KB (3) What tailored features do users expect? (4) What design specifications are ideal for usability? Each focus group session lasts 1 h with 5 segments: 1) an introduction to the study and basic functionalities of ALOHA; 2) the participants explore the ALOHA system freely for 20 min; 3) quantitatively assessements of usability using a modified System Usability Scale (SUS) [38]; 4) the users answer 4 open-ended questions to stimulate user thinking; and 5) at last, open discussions to gather user experience and feedback for improvements.

### Analysis of the data collected in usability assessment focus groups

We employed both quantitative and qualitative methods to analyze the data gathered from the focus group in each design iteration.

#### Quantitative analysis

To evaluate ALOHA’s usability quantitatively, we used the SUS that provides a quick view of the usability of the overall system [38]. The original SUS questions were created to evaluate the usability of systems, such as “*I think that I would like to use this system frequently*.” To make it more suitable for evaluating web-based application ALOHA, we simply replaced the word “system” with “website,” e.g., “*I think that I would like to use this website frequently.*” The SUS is, however, technology independent and has been used on evaluations of hardware, general software, websites, and mobile apps. The 10-item SUS questionnaire is based on a 5-point Likert scale and scales to a maximum score of 100 on the users’ impression of the usability of a system in general. A SUS score of 0 to 50 means the usability of the system is not acceptable, and a score of 50 to 70 means marginally acceptable. A score higher than 70 means the system’s usability is acceptable.

#### Qualitative analysis

We posted four open-ended questions before each usability assessment session: 1) “What other functions should be added to the website?”; 2) “Do you have any ideas or advice for this visualization?”; 3) “List the most negative aspect(s)”; and 4) “List the most positive aspect(s).” We then encouraged the participants to “think-aloud” and verbalize their experience interacting with ALOHA [39]. Participants were also encouraged to discuss other related issues, such as their perceptions and attitudes about using a system like ALOHA. With the participants’ consent, the focus group session was recorded and then transcribed.

The usability issues were identified and categorized by themes and heuristics. We used a 2-step process to qualitatively analyze the usability assessment sessions: 1) we collected an initial set of usability issues from users’ answers to the open-ended questions; and 2) we then analyzed the transcripts of the sessions to extract more usability issues from the conversations. All usability issues were encoded using themes derived from the thematic analysis [40] and mapped to usability heuristics defined in Gerhardt-Powals’ cognitive engineering principles with a focus on identifying any violations of the usability principles [41]. The usability heuristics contains 10 principles: 1) automating unwanted workload; 2) reducing uncertainty; 3) fusing data; 4) presenting new information with meaningful aids to interpretation; 5) using names that are conceptually related to function; 6) grouping data in consistently meaningful ways; 7) limiting data-driven tasks; 8) including in the displays only that information needed by the user at a given time; 9) providing multiple coding of data when appropriate; and 10) practicing judicious redundancy [41]. We followed a well-established process for the thematic analysis [40] commonly used in human-computer interaction projects consisting of 5 steps: 1) familiarizing with data (i.e., the answers to the open-ended questionnaire and the transcripts); 2) assigning initial annotation codes (i.e., a description of what has been said by the participants) to the data; 3) sorting (or grouping) codes into broader themes; 4) reviewing and refining the themes identified before; and 5) naming and describing each of the themes.

Through the thematic analysis, the usability issues reported by the participants were encoded by themes in each design iteration. We derived the usability themes based on the characteristics of the identified usability issues. Similar usability issues reported by different participants were grouped as a unique issue type.

More importantly, we extracted suggested usability improvements from the open-ended questions and the transcribed text from voice recording. These suggested improvements were analyzed and ranked by importance, which were used to inform the design choices in the next iteration.

## Results

### An evolving prototype of ALOHA

**Fig. 3:**
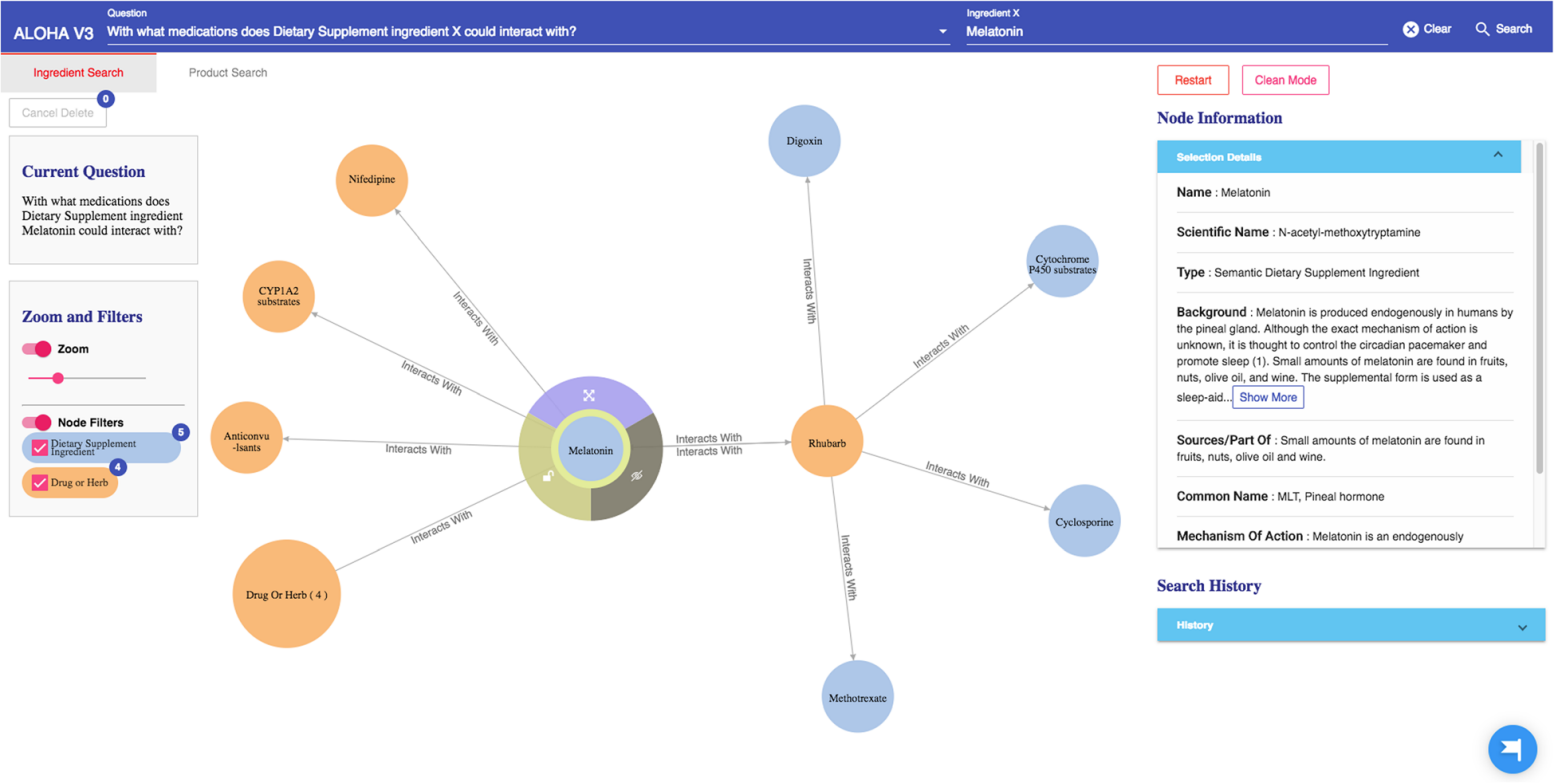
The main user interface of ALOHA

Compared to our previous ALOHA prototype in the first design iteration [28], Fig. 3 shows an evolved UI of ALOHA. The new UI consists of four parts: 1) a top bar for users to pick and enter DS questions of their interests based on a set of pre-defined question templates, 2) a canvas for graph-based interactive visualization, 3) a left group of information boxes including the current user question and various visualization options such as zooming and filtering, and 4) a right group of information boxes to show detailed information of the current selected node and search history.

#### Consumer questions

In the first design iteration, we analyzed a historical Yahoo Answers! dataset covering 2009 to 2014 (~ 3.8 million questions and 13.5 million answers posted by 339,193 users). Using the keyword “dietary supplement”, we found 157 questions related to DS in the “Health” category and primarily from the “Alternative Medicine” sub-category in Yahoo Answers!. Based on these questions, we identified the initial set of user needs. Combined with expert opinions and information contained in iDISK, we identified 7 question templates that consumers of DS are interested in. These questions are:

1. What is/are available product(s) containing DS ingredient X?
2. To which ingredient category does DS ingredient X belong?
3. Which LanguaL type does DS product with ingredient X belong to?
4. What is the background/origin of DS ingredient X? (24 out of 157; 15.4% of Yahoo Answers! posts, e.g., “*Phytochemistry - Thyme Thymol effects on human body as a dietary supplement?*”)
5. What are the common uses of DS ingredient X? (25 out of 157; 15.9% of Yahoo Answers! posts, e.g., “*Do amino acid dietary supplement help lose weight?*”)
6. What is/are the common adverse reaction(s) associated with DS ingredient X? (11 out of 157; 7.0% of Yahoo Answers! posts, e.g., “*I’ve heard that taking fennel as a dietary supplement helps with digestion, any negative side effects?*”)
7. With what medications does DS ingredient X could interact with? (3 out of 157; 1.9% of Yahoo Answers! posts, e.g., “*Can dietary supplements effect how a flu shot works?*”)

In the following two iterations, we extended the question templates and added three DS product related questions based on user feedback from the usability assessment session of the previous iteration:

1. What ingredients does Dietary Supplement product X contain?
2. What drug does Dietary Supplement product X interact with?
3. What diseases is Dietary Supplement product X effective for?

As the 3 new questions are all about DS products (rather than DS ingredients), we added a new search tab named “Product Search” to the top bar. Further, we also added a question type selection function to distinguish between ingredient or product related questions through switching the two tabs (i.e., “Ingredient Search” and “Product Search”) below the top bar.

In the “Ingredient Search” tab, as the questions are all focused on the relationships between a DS “ingredient X”, the top bar consists of 2 parts. As shown in Fig. 3, in the “Question” part (the left part of the top bar), the user first needs to choose a question template of her interest and then types the name of the ingredient in the “Ingredient X” part (the right column of the top bar). There is an autocomplete function, she only needs to enter a partial name of the ingredient. This functionality is similar in the “Product Search” tab except that it focuses on product-related questions.

#### Graph-based interactive visualization

As shown in Fig. 3, based on the user question, the system formulates high-level semantic queries using the Neo4j’s Cypher query language to query against the underlying iDISK knowledge base. The Neo4j graph database organizes data as nodes, relationships, and properties in a property graph model (PGM). Naturally, ontologically-structured data in iDISK can also be expressed in a PGM. The Neo4j Cypher is a declarative graph query language. One of the main advantages of the Cypher language is that its syntax is similar to the commonly used Structured Query Language (SQL) for relational databases but optimized to operate on PGMs. Nevertheless, the Neo4j’s query engine supports semantic queries with reasoning needs (i.e., leveraging the knowledge encoded in the ontology).

The query results (in responding to the specific user question) are then organized as a knowledge graph (i.e., nodes of concepts connected by edges indicating the relationships between nodes)—a relevant subgraph of the entire iDISK knowledge base. We built various convenient functions to make it easier for the users to explore the knowledge graph.

**Fig. 4:**
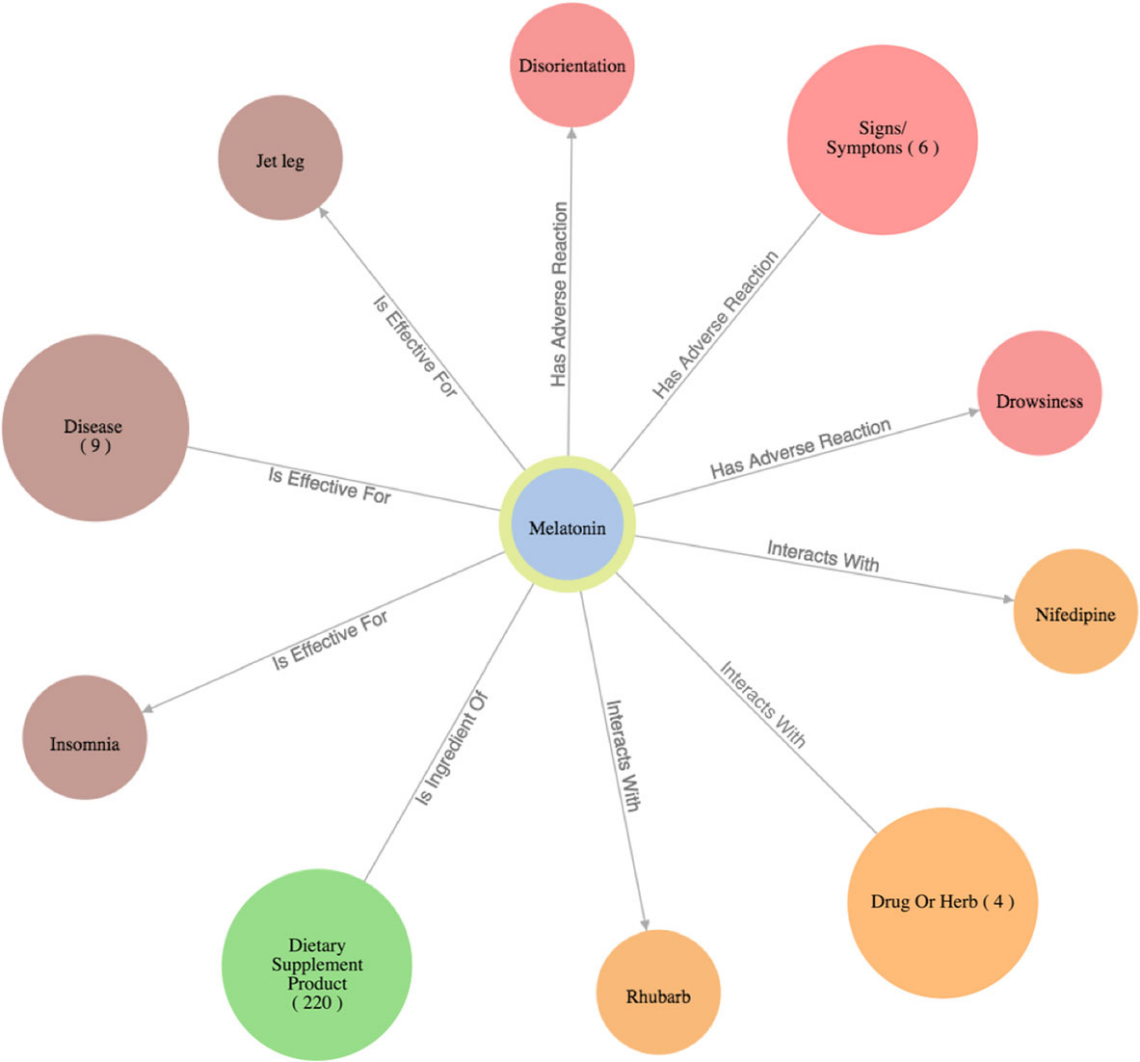
An example of both individual nodes and group nodes after expanding “Melatonin”

**Fig. 5:**
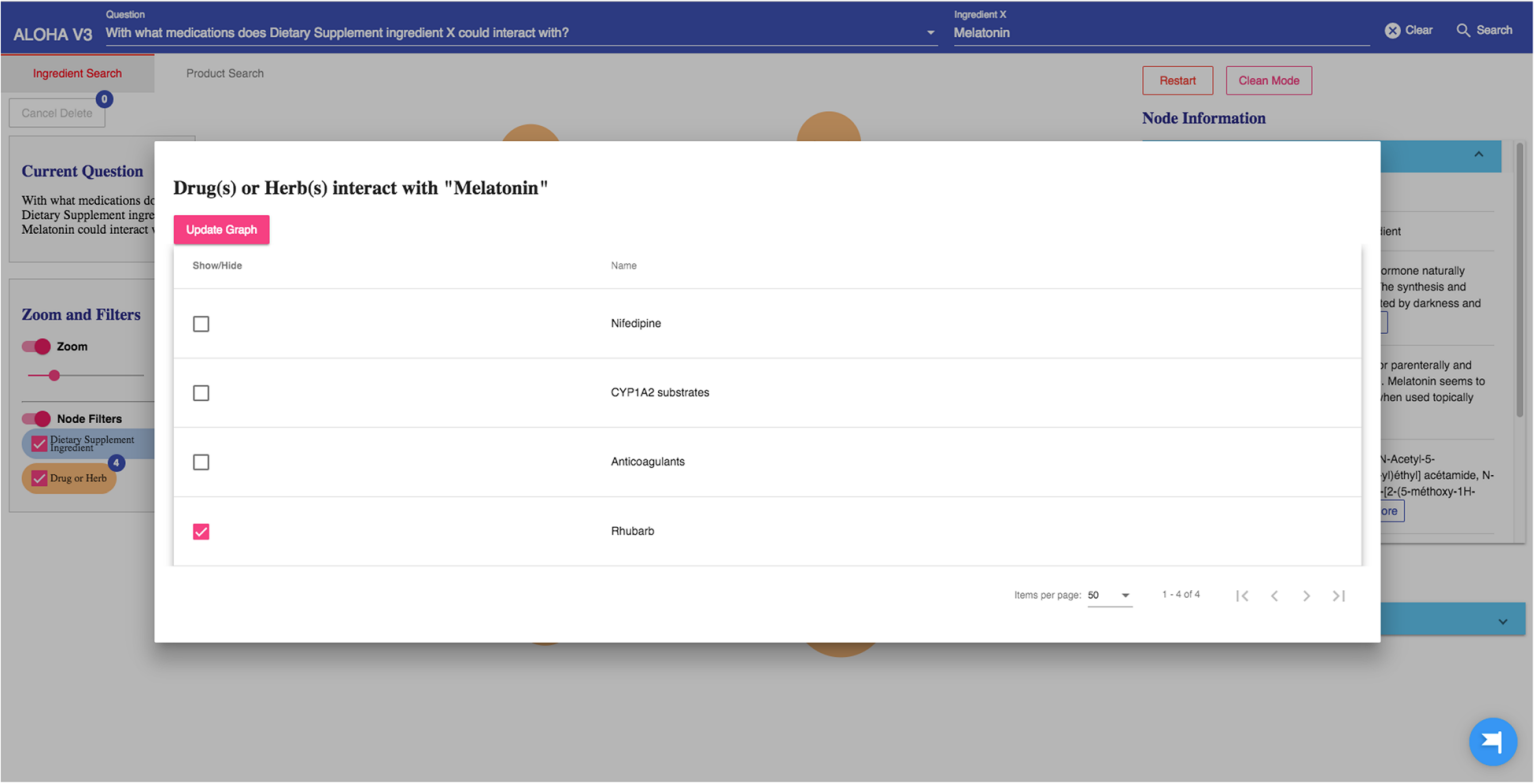
An interface for users to navigate the group nodes (i.e., the option to show or hide nodes of interest)

1. *Node expansion.* The node expansion function is the main functionality for users to explore the knowledge graph. As shown in Fig. 3, when a user double-clicks on a concept node or clicks on the purple expansion option button around the selected node, the system will expand the node and show all other nodes directly connected (i.e., 1-degree neighbors) to the selected nodes as well as the relationships between them. In the initial design, we restricted the number of nodes to be a maximum of 30 in each Cypher query result, considering that too many nodes displayed in one screen will be too crowded to navigate. However, in the following design iterations, we found that even a limit of 30 nodes for each Cypher query could result in hundreds of nodes after several node expansion operations. Further, some nodes have more than 30 neighboring nodes (e.g., “Ascorbic acid” and “Calcium” are respectively linked to 10,593 and 8,923 other nodes through corresponding relationships); and limiting the query result to only 30 nodes will result in incorrect answers to user’s question (i.e., showing a random set of 30 nodes that may not be of user interest). Thus, we re-implemented the node expansion functionality. As shown in Fig. 4, expanded nodes are grouped into 4 larger nodes (i.e., group nodes) by types (e.g. “DS Product”, “Disease”, “Signs/Symptoms”, “Drug/Herbs”). When a user clicks on a group node, as shown in Fig. 5, the user can choose to display or hide individual nodes by checking or unchecking the checkboxes of the nodes of interest in a pop-up table.

Every node, including individual node and group node, is color-coded based on its concept type. For example, as shown in Fig. 4, medications that interact with the specific DS ingredient are colored in orange, while DS ingredient nodes are colored in blue.

**Fig. 6:**
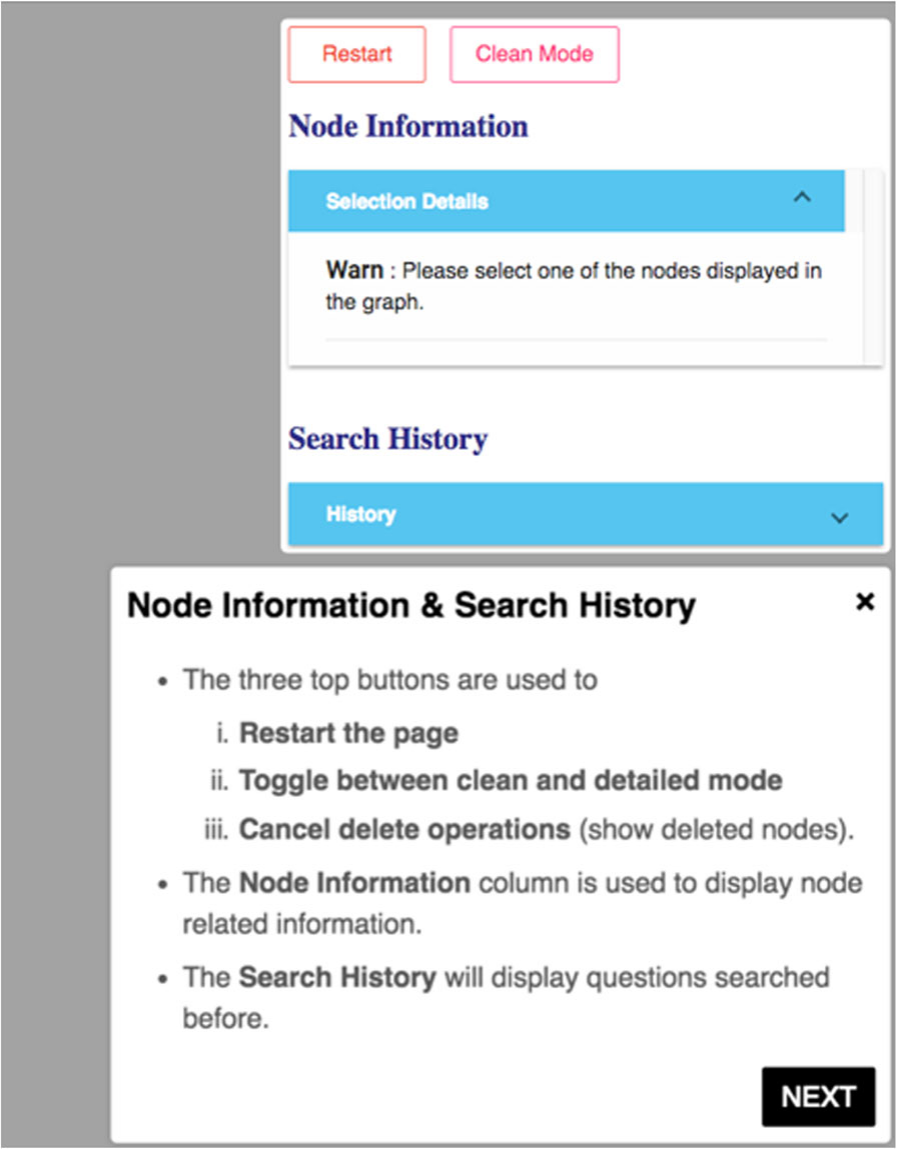
An example of an interactive ALOHA tutorial built with Help Hero

2) *Tutorial.* By employing HelpHero [42], a tool for creating interactive, easy-to-follow web application tours, we created four tutorials to help users get started and learn new features of ALOHA quickly. At the first time when a user enters ALOHA, the “Brief Introduction” tutorial will appear automatically and briefly introduce the main functionalities of ALOHA, such as the search bar and node information boxes. As shown in Fig. 5, a blue flag icon on the right bottom corner of the screen allows the end users to re-access the tutorials at any time. Figure 6 shows an example of the tutorials. Currently, there are two video tutorials and two tour tutorials.
3) *Node information box on the right sidebar.* When a node is selected, more information about the node such as the background and safety information of a DS ingredient is shown on the right side of the screen. Compared to the previous version of ALOHA, we removed a number of unnecessary information based on user feedback and shortened the length of long text description by utilizing a “Show More / Hide” functionality, to reduce the cognitive load on end-users.

### Usability assessment focus group results

In the first design iteration in our previous work [28], the average SUS score of the initial ALOHA prototype was marginally acceptable (63.75 ± 7.2). The focus group participants have also indicated very different experience when using ALOHA. Overall, 23 distinct usability issues were reported by the participants; and our analysis grouped the 23 usability issues into 7 themes (Table 1): 1) lack of functionalities, 2) unnecessary information, 3) incomplete functionality, 4) unclear information presentation, 5) incomplete information, 6) lack of information, and 7) unintuitive information. The majority of these usability issues are related to lack of information or functionalities. We also mapped these distinct usability issues to the 10 usability heuristics described in Gerhardt-Powals’ cognitive engineering principles. The most frequent usability heuristics are a) reducing uncertainty and b) including in the displays only the information needed by the user at a given time.

**Table 1:** Usability issues identified in the first design iteration’s usability testing focus group

Theme	Usability Issue	Heuristic	Number of participants reporting the issue (*n* = 9)
Incomplete functionality	Some weird characters, unreadable Unicode characters	Reduce uncertainty	1/9
	There are two across signs work the same way, one of them should be modified	Reduce uncertainty	1/9
	Two graphs in the same page separate too far away	Reduce uncertainty	1/9
	Can not read the full text content of some questions	Reduce uncertainty	1/9
	Can not read the full name on the circles	Reduce uncertainty	1/9
Lack of functionality	Users can not hide information after they have looked at them	Include in the displays only that information needed by the user at a given time	1/9
	Users can not remember the question they asked	Reduce uncertainty	2/9
	Don’t know how to start to use the system	Reduce uncertainty	2/9
	Expect more information when hovering on nodes	Practice judicious redundancy	1/9
	No alert message for error or null value	Reduce uncertainty	2/9
	Questions should be able to start from everywhere (products, ingredient, etc.)	Provide multiple coding of data when appropriate	1/9
	Users need go through every circle to figure out the anwser of a question	Group data in consistently meaningful ways	1/9
	URL is fixed, users can not return to the previous page	Automate unwanted workload	1/9
Lack of information	No sufficient information for some relationships(like “effects_on”, need more details)	Provide multiple coding of data when appropriate	1/9
Unclear information presentation	Links overlap and relationships are hard to be read	Reduce uncertainty	1/9
	Nodes’ font is difficult to be read	Reduce uncertainty	2/9
	Small finder icon, it is hard for user to notice it	Present new information with meaningful aids to interpretation	1/9
Unintuitive information	Questions are not easy for laypeople to understand	Present new information with meaningful aids to interpretation	1/9
Unnecessary information	Preferred names and scientific names are unnecessary nodes	Fuse data	1/9
	Redundant information for some questions’ results	Include in the displays only that information needed by the user at a given time	2/9
	Show too much information without limitation	Include in the displays only that information needed by the user at a given time	3/9
	Unnecessary information (Number in the parentheses) in right column	Include in the displays only that information needed by the user at a given time	1/9
	Unnecessary information (UMLS Semantic Type) in right column	Include in the displays only that information needed by the user at a given time	1/9

The usability improvements suggested by the participants were consistent with the identified usability issues. These proposed improvements included: 1) removing unnecessary information; 2) grouping similar information; 3) changing the expression of data to be easier for laypeople to consume; 4) improving readability; and 5) adding functions. Table 2 lists selected suggested improvements and our corresponding actions for each design iteration.

**Table 2:** Selected important improvements suggested in the first design iteration

Improvement Category	Improvement Action
Removing unnecessary information	Remove number in the parentheses
	Remove UMLS Semantic Type
Grouping similar information	Group questions with same types
	Group same types of information together
Changing the expression of data to be easier for laypeople to consume	Find layperson questions that someone who is using the database would probably ask
Adding functions	Add toggle function for nodes to display information
	Add tutorial
Improving readability	Change the color and size of texts in the nodes

In the second design iteration, we improved some of the existing features but primarily added many new features according to reported usability issues. In the usability assessment session of the second design iteration, we recruited 8 participants where 4 of them attended the usability assessment session in the first iteration as well. The total number of usability issues (Table 3) reported by the participants decreased. The total number of usability issues reported was 22, and there were 13 distinct issues after grouping. The usability themes revealed were a) readability, b) long response time, c) information presentation, d) lack of functionalities, and e) incomplete functionality.

**Table 3:** Usability issues identified in the second design iteration’s usability testing focus group

Theme	Usability issue	Heuristic	Number of participants reporting the issue (*n* = 8)
Readability	The words in the tutorial are too small	Reduce uncertainty	2/8
	The word on the line is too close to the line	Reduce uncertainty	1/8
Long response time	The loading time for queries is too long	Reduce uncertainty	5/8
Information presentation	The web application layout design is initially not easy to understand	Present new information with meaningful aids to interpretation	1/8
	The search box should be on the top	Automate unwanted workload	2/8
	The text in the node should be maintained by word level	Automate unwanted workload	1/8
	Some nodes will be out of the viewport after node dragging	Reduce uncertainty	1/8
	It is hard to find the most important information from the result	Include in the displays only that information needed by the user at a given time	1/8
Lack of functionalities	There should be a way to cancel delete node operation	Automate unwanted workload	2/8
	Search history is needed	Automate unwanted workload	2/8
Incomplete functionality	The tutorial should be more comprehensive	Automate unwanted workload	2/8
	Sometimes the disease node can’t expand	Reduce uncertainty	1/8
	The arrow should be a double direction	Reduce uncertainty	1/8

However, the SUS score decreased to 52.2 ± 11.0. After further analysis of the usability testing results, we found that the newly added functionalities enhanced and enriched the ALOHA application but also increased the complexity of the overall system. Simply adding new functionalities or changing existing features without due consideration could bring severe usability issues and impair users’ experience of using the application. Further, for the second design iteration, the suggested usability improvements (as shown in Table 4) changed to mostly focused on 1) refining the user interface, 2) improving existing functions, 3) adding auxiliary functionalities to make ALOHA more usable, and 4) improving readability.

**Table 4:** Selected important suggested improvements from the second design iteration’s usability assessment session

Improvement Category	Improvement Action
Improving readability	Change text size in the tutorial
Improving user interface	Move the search box to the top of the page
	Modify the layout of the page
Improving functions	Tune the Cypher queries and Neo4J database to reduce query time
	Make a more comprehensive tutorial
Adding auxiliary functions	Add “search history” function
	Add “cancel delete operation” function

Learning from the previous design iterations, in the third design iteration, we focused mostly on improving the existing functionalities. The SUS score increased significantly to 64.4 ± 7.2, which means that the usability of ALOHA increased compared to previous versions even with more complex and powerful functionalities.

In the third design iteration, we recruited 8 participants where 5 out of the 8 participants attended the previous design session. The total number of usability issues reported was 22; 10 distinct issues (Table 5) remained after grouping, and most of these issues were minor. The usability themes revealed were a) lack of instruction, b) system complexity, c) incomplete functionality, d) long response time and e) unclear information presentation. Compared to the second design iteration, there were no new features requested from users. Table 6 lists the suggested usability improvements.

**Table 5:** Usability issues identified in the third design iteration usability testing focus group

Theme	Usability Issue	Heuristic	Number of participants reporting issues (*n* = 8)
Lack of instruction	Lack of instructions for the node buttons	Reduce uncertainty	2/8
System complexity	Need to watch the tutorial first before using the system	Reduce uncertainty	3/8
Incomplete functionality	Search history should not contain duplicates	Group data in consistently meaningful ways	1/8
	Search history should be categorized by type	Fuse data	3/8
	Sometimes the lines between nodes disappear after filtering	Reduce uncertainty	1/8
	The zoom button is not moving with the mouse pointer	Reduce uncertainty	1/8
	The number of provided questions is not enough	Provide multiple coding of data when appropriate	1/8
	The “Brief introduction” tutorial appears every time	Automate unwanted workload	1/8
Long response time	The reaction time for product search autocomplete is too long	Reduce uncertainty	2/8
Unclear information presentation	It is a little hard to understand the search results	Present new information with meaningful aids to interpretation	2/8

**Table 6:** Selected important suggested improvements from the third design iteration’s usability assessment session

Improvement Category	Improvement Action
Revising existing functions	Categorize search history
	Add more questions for searching
	Improve the performance of autocomplete function using database
Polishing user tutorials	Make the tutorial more comprehensive

## Discussion

The use of DSs (e.g., vitamins, minerals, botanical extracts, and protein powders) is common around the globe, although consumers have limited knowledge of their safety and effectiveness. In this study, we conducted two design iterations to further improve our prototype interactive visualization system, ALOHA [28], based on a well-integrated DS knowledge base—iDISK. We followed user-centered design principles during these design iterations, leading to a more user-friendly and useful application.

### Two use cases demonstrating the utility of ALOHA

The utility of ALOHA can be demonstrated through two use cases.

### Use case 1: searching for drugs that interact with a DS product “Sleepaid”

**Fig. 7:**
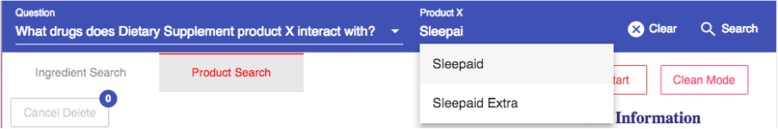
A user searches for a question: “What drugs does Dietary Supplement product Sleepaid interact with?”

A user has recently encountered some sleeplessness problems, and she started to take “Sleepaid”—a DS product used for relief of occasional sleeplessness. However, she is also taking other medications at the same time, and she is curious about whether “Sleepaid” has some interactions with the medications she is taking. So, the user decides to give ALOHA a try. First, as shown in Fig. 7, she chooses the “*Product Search*” under the top search box and asks the question “*What drugs does Dietary Supplement product Sleepaid interact with?*” She receives the query result in few seconds visualized as a graph.

**Fig. 8:**
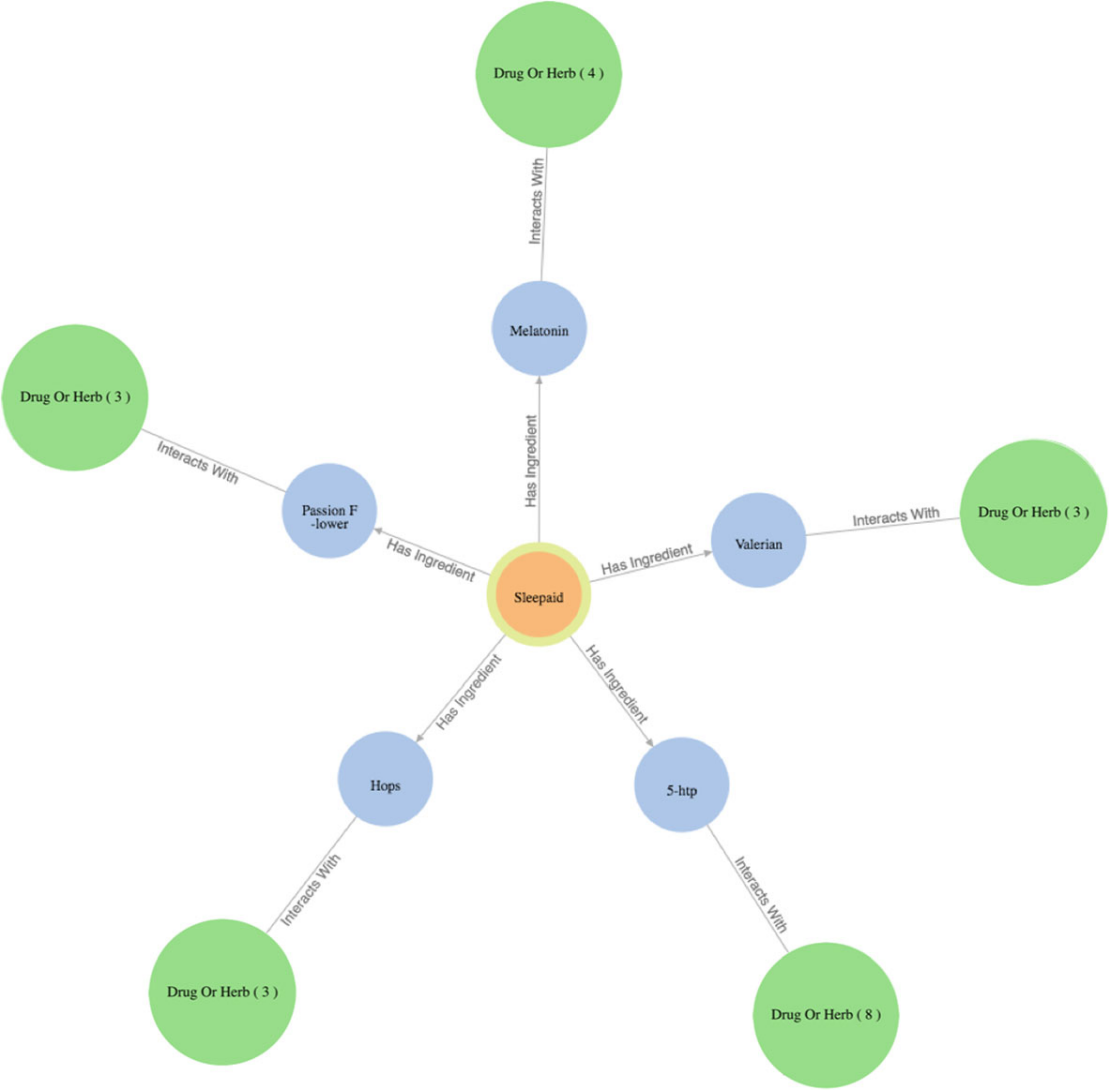
Query results as an interactive graph-based visualization the for question “What drugs does Dietary Supplement product Sleepaid interact with?”

**Fig. 9:**
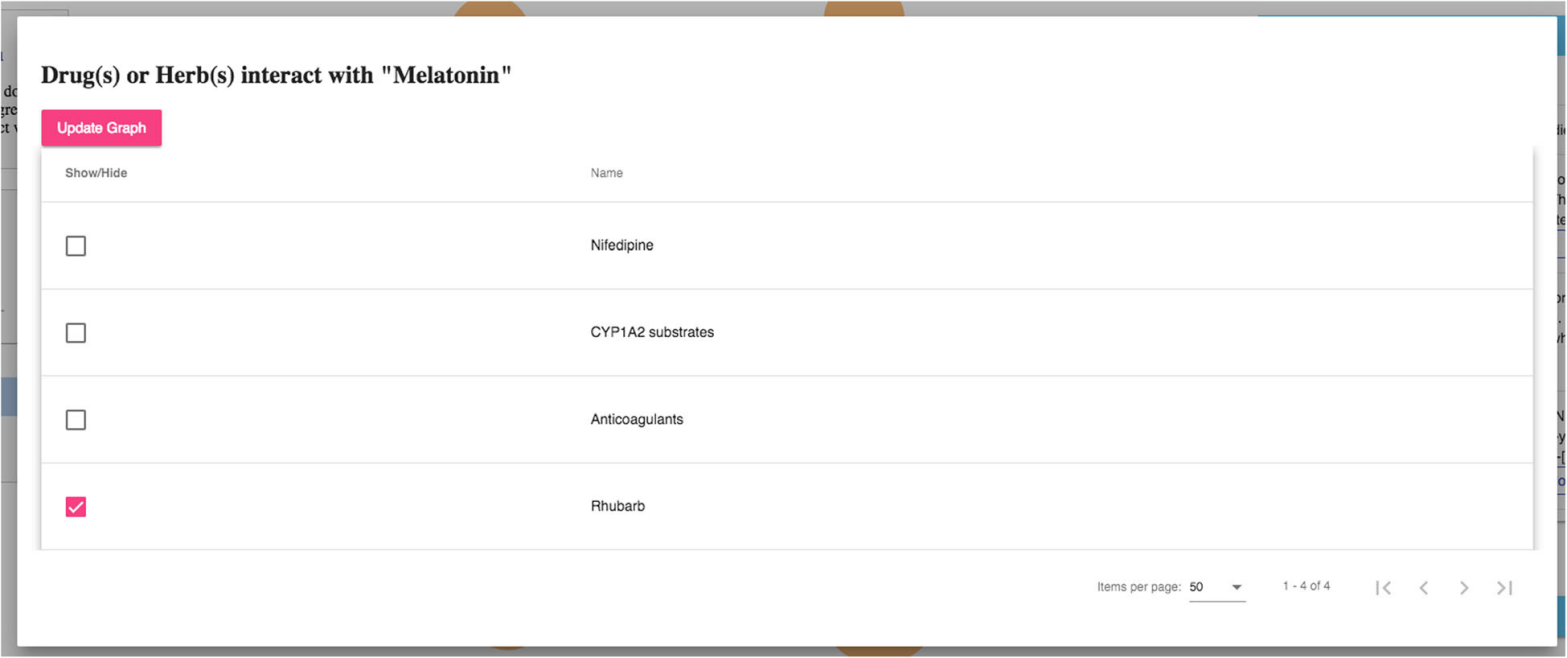
A list of 4 drugs or herbs that can interact with Melatonin

**Fig. 10:**
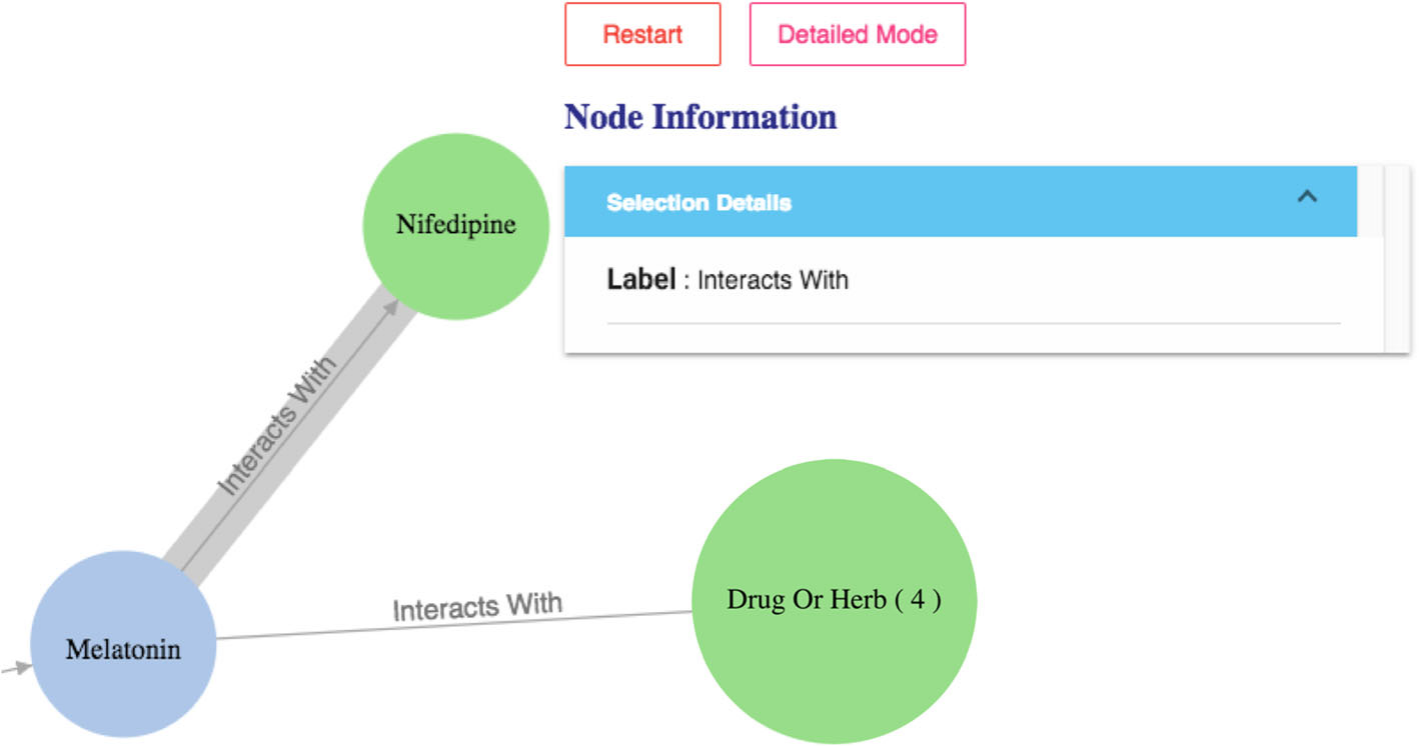
The “interacts with” relationship between Nifedipine and Melatonin

As shown in Fig. 8, Sleepaid contains 5 DS ingredients (i.e., 5-htp, Melatonin, Passion flower, Valerian, and Hops), and each ingredient can potentially interact with different drugs. The user knows about *Melatonin* as the main ingredient in Sleepaid; and found from ALOHA that *Melatonin* can interact with 4 drugs/herbs. She then clicks the “*Drug/Herb*” group node that is linked to Melatonin and a table pops up showing the 4 drugs that *Melatonin* can interact with. As shown in Fig. 9 and Fig. 10, she found that *Nifedipine*, a drug she is taking for her high blood pressure, is in the list, which makes her worry about the potential adverse effects. Thus, she wants to find a replacement of *Melatonin* as well as the DS product “Sleepaid”.

### Use case 2: finding a replacement of “melatonin”

**Fig. 11:**
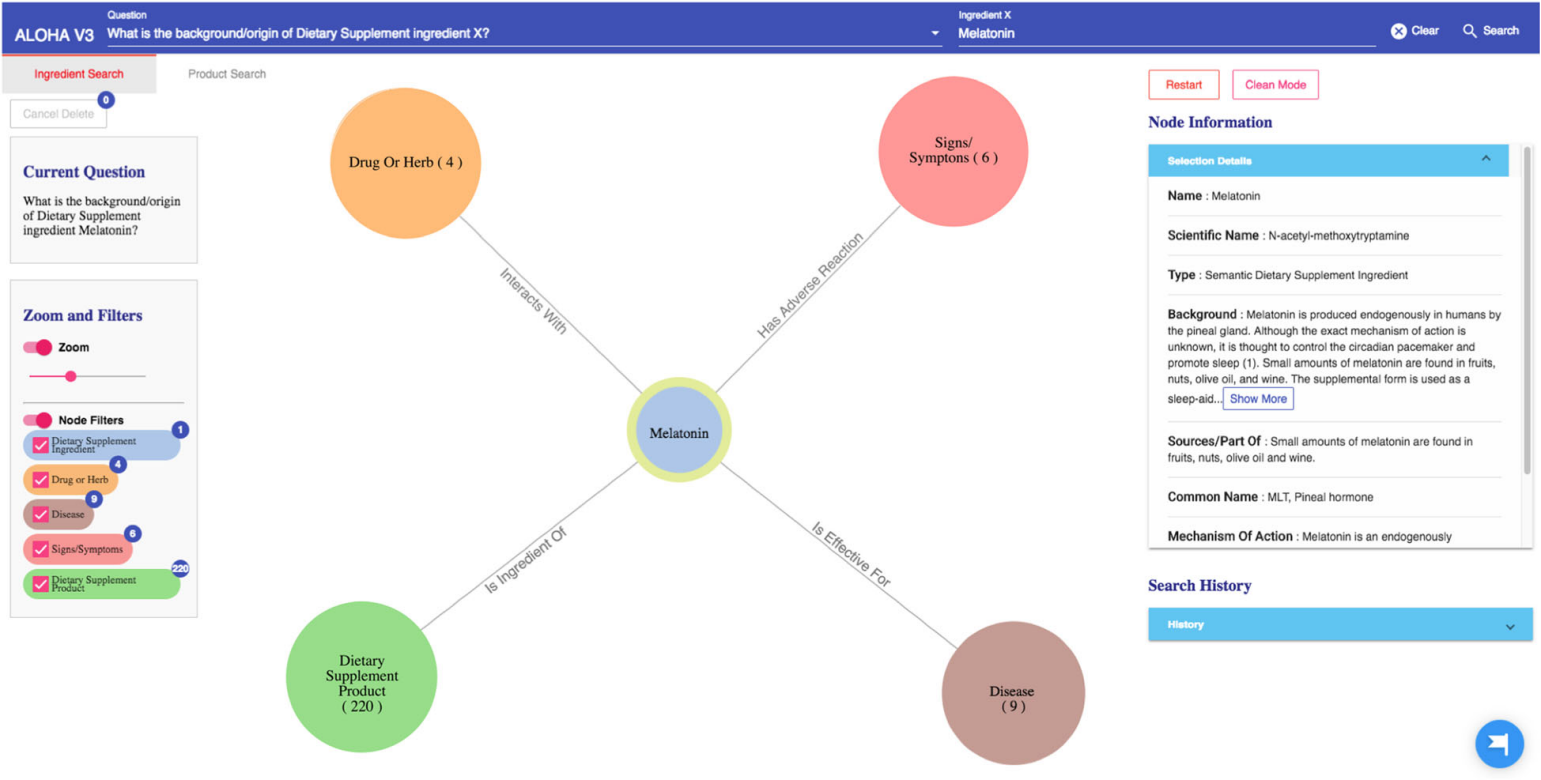
The query result of question “What is the background/origin of Dietary Supplement ingredient Melatonin?”

**Fig. 12:**
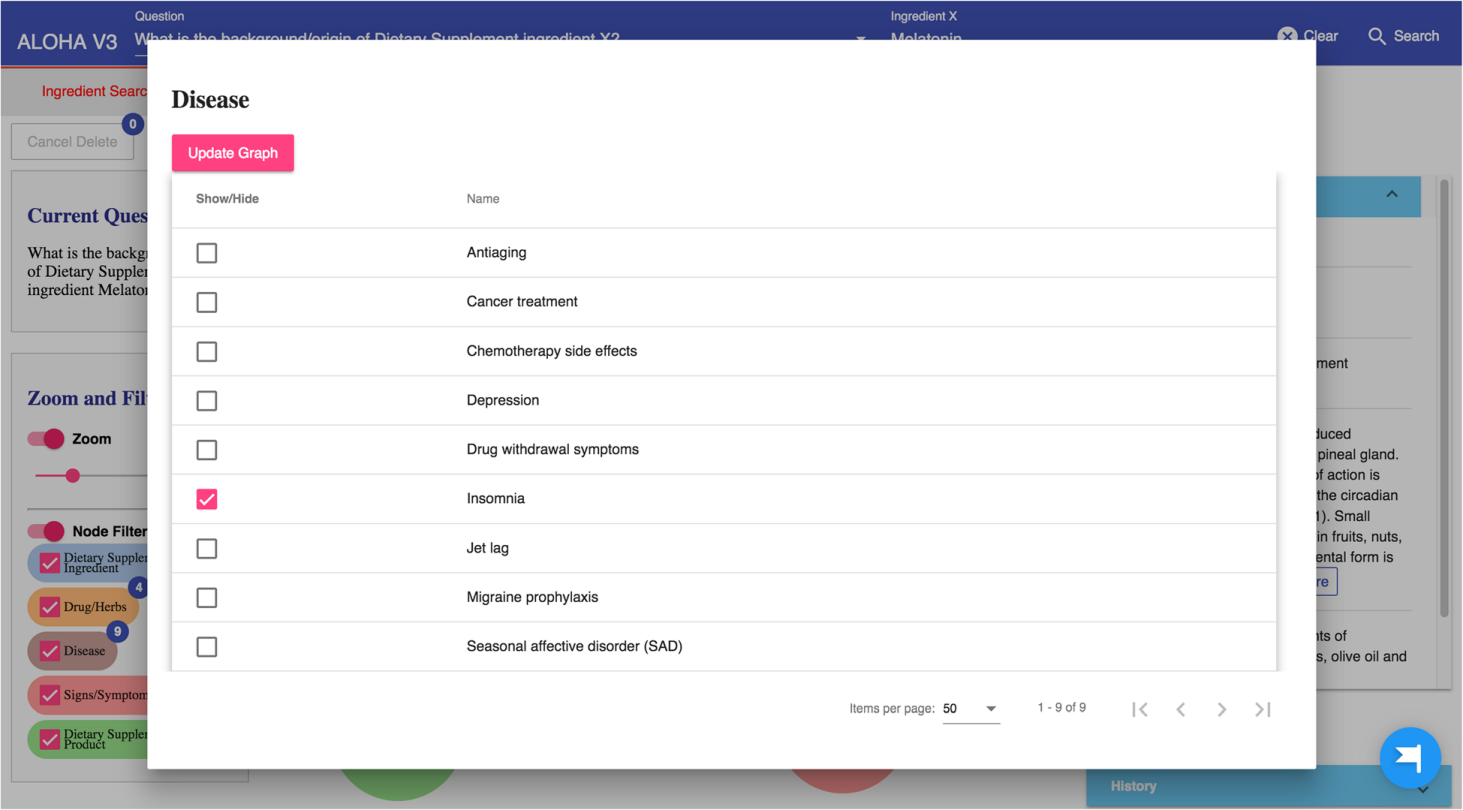
A list of diseases for which Melatonin is effective

**Fig. 13:**
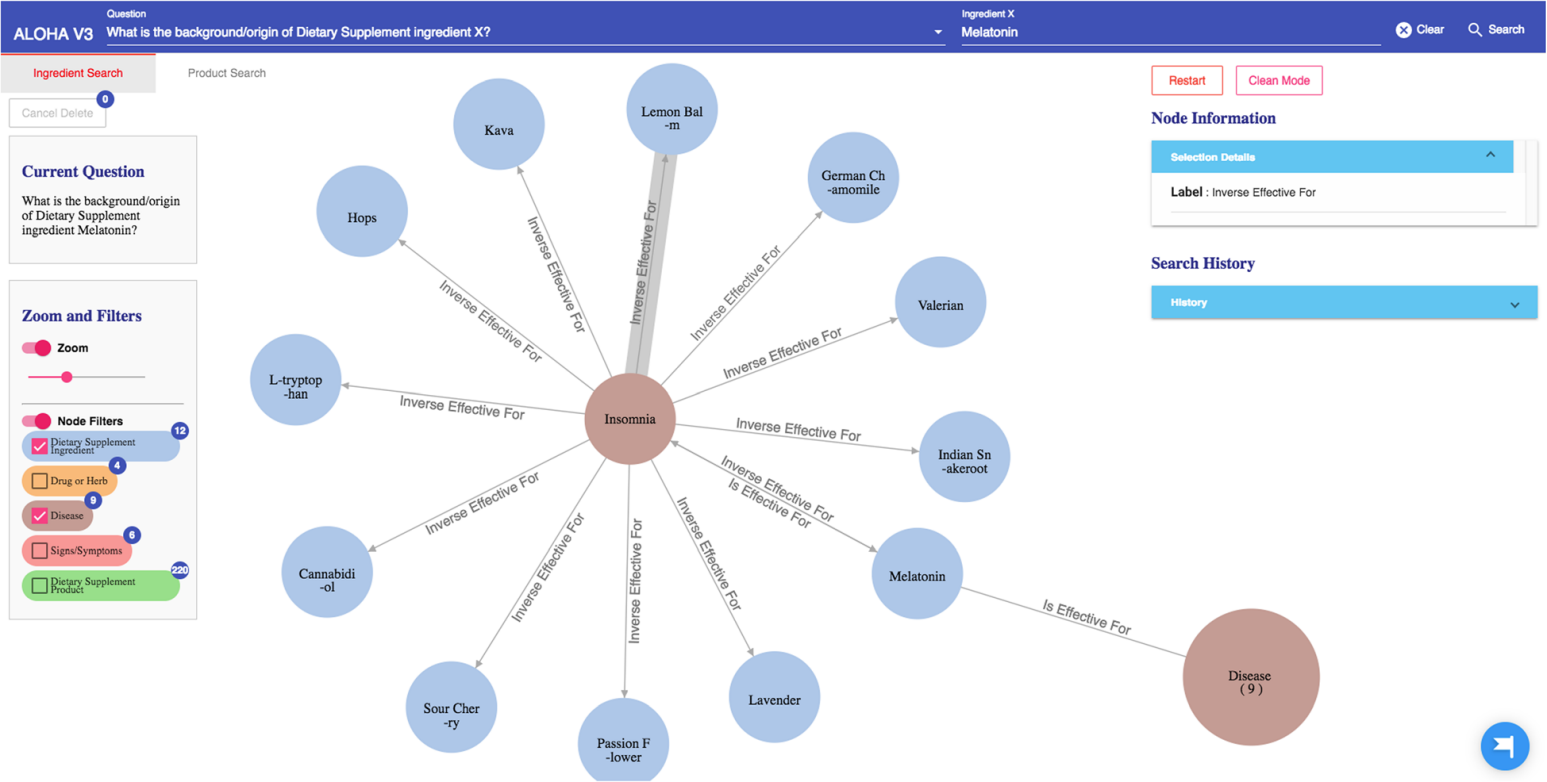
A list of DS ingredients which are effective for sleeplessness

Because of the observed interaction between *Melatonin* and *Verapamil*, the user decides to learn more about *Melatonin* and possibly find a replacement of *Melatonin* to address her sleeplessness issue. She first explores the DS ingredient *Melatonin* in ALOHA. As shown in Fig. 11, she chooses the “Ingredient Search” tab and enters the question “*What is the background/origin of Dietary Supplement ingredient Melatonin?*”. The system responds with a graph of 4 group nodes linked to *Melatonin*, and the “Node Information” box shows the background and safety information about *Melatonin*. After reading the information about *Melatonin*, the user becomes curious about what diseases *Melatonin* is effective for. Therefore, she clicks the brown disease group node; and the system shows her a list of 9 diseases for which *Melatonin* is effective. As shown in Fig. 12, the user notices the disease “sleeplessness,” which is one of her current health problems. Hence, the user chooses “sleeplessness” to see what other DS ingredients might be effective for helping with sleeplessness. The user expands the “sleeplessness” node but hides other types of nodes that are not of interest to her through the “Filter” function, as shown in Fig. 13. After checking the effectiveness rating (part of the “Node Information” as shown in Fig. 13) of each DS ingredient for sleeplessness, the user finds only *Lemon Balm*, *Melatonin* and *Valerian* are possibly effective. Subsequently, the user decides to explore more about *Lemon Balm* and *Valerian* to determine which DS ingredient is safer and more effective considering her current health issues.

### Lessons learned from an iterative user-centered design process in developing consumer-facing health applications

The lessons learned from the three design iterations are valuable to guide not only future developments of ALOHA but also other consumer-facing, knowledge graph-based online health information systems. Through the three usability testing focus groups, a number of usability issues were revealed with specific action points to improve both the usability and utility of ALOHA. Nevertheless, simply adding all functionalities requested by end-users without due consideration could bring severe usability issues and impair user experience. The SUS score in our second design iteration dropped significantly to 52.2 ± 11.0 from 63.75 ± 7.2 mainly due to these issues. For example, based on user requests, we added a number of tutorials hoping that they will help users quickly get a sense of the different functionalities of the system. However, in the usability evaluation focus group of the second design iteration, a number of participants ignored the newly added tutorials. When asked, they expressed that “*I am confident that I can learn how to use the system myself.*” However, compared to other resources that users used to find relevant health information such as Google search engine and WebMD, a graph-based visualization tool is significantly different; and thus, it is not easy for users from the general public to get familiar with quickly. There were also many usability issues in the tutorials, which impaired the user experience. As shown in Table 3, two of the eight participants thought that the texts in the tutorials are too verbose and the size of the text font is too small; so they chose to ignore the tutorials all together. Further, two of the eight participants thought that the tutorial was not comprehensive enough to cover all features of ALOHA. Thus, more work is needed to refine these tutorials.

We also revised a number of existing functionalities, especially improved a number of areas around the interactive visualization. However, the response time for ALOHA increased significantly as we introduced the new visualization mechanisms. For the ALOHA prototype in the first design iteration, we set a constraint (i.e., a maximum of 30 nodes) to each Cypher query to reduce the number of nodes rendered in the visualization to make the graph more clear. In the second design iteration, we removed the restriction as we want to get a more accurate answer for each query. Nevertheless, some queries resulted in a large number of nodes (i.e., more than 1000 with a long query execution time) that filled the entire screen making the information unreadable. To solve this problem, we introduced a new visualization mechanism, where we grouped nodes of the same semantic type to reduce the total number of nodes on the screen. This new design not only simplified the user interface but also reduced the query execution time that ultimately improved overall user experience.

## Conclusions

In sum, a user-centered design process enabled us to create a user-friendly web-based application, ALOHA, for the general public, especially DS consumers, to explore DS knowledge relevant to their needs, through an iterative development process. Moreover, our study showed that graph-based interactive visualization is promising in helping DS consumers explore complex health concepts quickly and can potentially lead to a new way of finding and consuming health information online. This is significant as recognized by many health behavior theories such as the integrated behavior model, accessing to adequate health information activates individuals’ participation in self-care and leads to healthy life-style [43]. Conversely, inaccessibility to adequate health information is associated with serious health risks [44]. In the United States, 72% of adults seek health information online [45]. Nevertheless, typical consumers cannot translate the vast amounts of online information into usable knowledge, nor gauge its quality [46]. Information access barriers include overload and disorganization, lack of user-friendliness, and inconsistencies [47]. A novel knowledge exploration mechanism such as ALOHA maybe appealing to the general public and can serve as a template for developing consumer-facing, evidence-based (i.e., supported by scientific literature) knowledge graphs in many other health and disease domains. Nevertheless, future work is warranted in ALOHA to address other online health information issues such as translating and linking the scientific terms in iDISK into consumer language. Further, a more formal assessment of ALOHA’s visual interface is needed to demonstrate its effectiveness, for example, through comparing end-users’ knolwege gains between text comprehension and visual comprehension of the same information.

## Abbreviations

ADE: Adverse Drug Event
ALOHA: dietAry suppLement knOwledge grapH visuAlization
API: Application Programming Interface
CSV: Comma-separated Values
DDI: Drug-drug Interaction
DS: Dietary Supplement
DSLD: U.S. Dietary Supplement Label Database
FDA: Food and Drug Administration
HTTP: Hypertext Transfer Protocol
iDISK: integrated DIetary Supplement Knowledge base
KB: Knowledge Base
LNHPD: Licensed Natural Health Products Database
NHPID: Canadian Natural Health Product Ingredient Database
NLM: National Library of Medicine
NM: Natural Medicines
PGM: Property Graph Model
RESTful: Representational State Transfer-ful
RRF: Rich Release Format
SQL: Structured Query Language
SUS: System Usability Scale
UCD: User-Centered Design
UMLS: Unified Medical Language System
UX: User Experience
